# Higher melatonin in the follicle fluid and MT2 expression in the granulosa cells contribute to the OHSS occurrence

**DOI:** 10.1186/s12958-019-0479-6

**Published:** 2019-04-12

**Authors:** Yiran Li, Lanlan Fang, Yiping Yu, Hao Shi, Sijia Wang, Yanjie Guo, Yingpu Sun

**Affiliations:** Reproductive Medical Center, The First Affiliated Hospital of Zhengzhou University, 450052 Zhengzhou, People’s Republic of China Zhengzhou No. 1 construction east road, He’nan Province, China

**Keywords:** Melatonin, Melatonin receptor 2, Follicular fluid, Ovarian hyperstimulation syndrome, Controlled ovarian hyperstimulation

## Abstract

**Background:**

Ovarian hyperstimulation syndrome (OHSS) is a common and severe complication for patients undergoing IVF/ICSI-ET. Melatonin widely participates in the regulation of female reproductive endocrine activity. However, whether melatonin participates in the progression of OHSS is largely unknown. This study aims to identify the predictive value of follicular fluid (FF) melatonin for OHSS establishment and the underlying mechanism.

**Methods:**

All participants of this case-control study were enrolled at the Reproductive Medicine Center located in the First Affiliated Hospital of Zhengzhou University in China from January to October in 2017. Quantitative real-time PCR and western blot were used to examine the mRNA and protein levels. Primary granulosa cells were extracted and cultured for in vitro studies. Melatonin concentration was measured by ELISA. Logistic analysis and receiver-operating characteristic (ROC) curves were used to evaluate the predicting value of melatonin on OHSS occurrence.

**Main outcome measures:**

The expression level of melatonin receptor 2 (MT2), P450 aromatase cytochrome (aromatase), vascular endothelial growth factor (VEGF), and inducible nitric oxide synthase (iNOS) mRNA in human primary granulosa cells. The concentration of melatonin in FF. The predicting value of melatonin on OHSS and the cut-off value of the prediction.

**Results:**

FF melatonin concentrations were significantly higher in patients with OHSS compared to non-OHSS group (35.94 ± 10.18 ng/mL vs 23.93 ± 10.94 ng/mL, *p*<0.001). The expression of MT2 mRNA (*p* = 0.0459) and protein in granulosa cells was also significantly higher in the OHSS group. When using a cut-off level of 27.52 ng/ml, the sensitivity and specificity of FF melatonin to predict OHSS was 84.6 and 74.0%, respectively (*p* < 0.0001). We also found that melatonin could up-regulates aromatase mRNA, VEGF mRNA expression and down-regulates iNOS mRNA expression in the granulosa cells**.**

**Conclusion:**

OHSS patients have higher melatonin in the FF as well as higher MT2 expression in the granulosa cells. The melatonin in FF might be used as an effective predictor for the occurrence of OHSS.

## Introduction

Melatonin (N-acetyl-5-methoxytryptamine) is a compound extracted from pineal glands and peripheral nerve [1]. Its expression is affected by light and dark stimulation, in accordance to circadian rhythm [2]. Previous investigations about melatonin mainly focused on its broad-spectrum free radical scavenging capacity and antioxidant capacity in the reproductive system [3]. Melatonin participates in the regulation of hypothalamic-pituitary-gonadal axis function and reproductive endocrine activity [2, 4]. Melatonin receptor 1 (MT1) and 2 (MT2), belonged to G protein–coupled receptors have been successfully cloned in humans [5–7]. Earlier studies have demonstrated that MT1 and MT2 are widely distributed in the female reproductive system, such as the uterus, ovaries, the epithelial cells of the mammary gland, and the granulosa cells [8]. Furthermore, according to a single-nucleotide polymorphisms (SNP) study, rs10830963 in MT2 gene may predispose the polycystic ovarian syndrome (PCOS) in the Chinese population [9], which indicate that MT2 might regulate follicle development and ovary reactivities. Since the high response and sensibility, the PCOS patients who are under controlled ovarian hyperstimulation (COH) have a higher risk of ovarian hyperstimulation syndrome (OHSS) [10]. In addition to the reproductive system, MT2 has also been found in the blood vessels, potentially allowing melatonin to induce vasodilatation which is the main feature of OHSS [11, 12].

OHSS is a common and severe complication for patients who undergo COH [13, 14]. Serious complications of OHSS may present as ascites, hydrothorax, electrolyte imbalance, hypovolemic shock and even threatening life [12]. Currently, several factors like rennin angiotensinogen system (RAS), vascular endothelial growth factor (VEGF), interleukins, nitric oxide (NO), tumor necrosis factor (TNF) and estrogens have been identified as causative agents of OHSS [15, 16]. High level of estrogen and high vascular permeability are the main characteristics of OHSS. As a key rate-limiting enzyme in the transformational process of androgen to estrogen, aromatase can mediate the production of estrogen [17]. While VEGF and NO can regulate the vascular permeability through angiogenic cytokines and inflammatory factors [18, 19]. In addition, inducible nitric oxide synthase (iNOS) promotes NO production and activates macrophages to regulate vascular immune inflammation [19, 20]. However, the pathogenesis of OHSS is still not totally illustrated. Thus, how to predict and prevent the occurrence of OHSS at an early stage is still a great challenge.

Recent studies mainly focused on the effects of melatonin on ovarian function and the oocytes [21–25]. The antioxidant effects of melatonin can protect oocytes from the damage of oxidative stress [23]. In addition, the effects of melatonin on the oocytes can be mediated by its receptors [24, 25]. Follicular fluid (FF) melatonin concentrations are highly associated with both quantity and quality of oocytes and the ovarian reserve [26]. While higher ovarian reserve and more retrieved oocytes are high risk factors of OHSS [12]. Considering MT2 has also been found in the blood vessels, potentially allowing melatonin to induce vasodilatation which is the main feature of OHSS, it is hypothesized that melatonin and its receptors participate in the OHSS occurrence. However, the expression and function of melatonin and its receptors in the OHSS are still unknown. Thus, the aims of our present study were to investigate the role of melatonin in the OHSS occurrence and the underlying molecular mechanism. Our study was also designed to test the relationship between FF melatonin and other parameters and whether FF melatonin level could be a potential predictor for the occurrence of OHSS.

## Materials and methods

### Patients

A total of 57 patients participated in this investigation. All participants enrolled at the Reproductive Medicine Center located in the First Affiliated Hospital of Zhengzhou University in China from January to October in 2017. Study protocols were approved by the Zhengzhou University Research Ethics Board and all participants provided informed consent prior to enrollment. The criteria for included patients were as follows:1) age between 20 and 35; 2) BMI between 18 and 25 kg/m^2^; 3) male infertility or tubal infertility; 4) regular menstrual cycles; and 5) without comorbidities such as deranged thyroid function or diabetes. The exclusion criteria were: 1) PCOS, endometriosis or diminished ovarian reserve patients; 2) patients possess hydrosalpinx; 3) patients had a chromosomal abnormality. Patients enrolled in this study were grouped into OHSS or non-OHSS according to their clinical manifestation based on the Golan classification system [27]. Ultimately, a total of 26 OHSS and 31 non-OHSS patients were recruited.

### IVF/ICSI-ET protocols

The standard long protocol for IVF/ICI-ET was treated to all patients. Primarily, gonadotropin-releasing hormone agonist (triptorelin acetate; Ferring AG, Switzerland) was injected for downregulation. Then recombinant FSH (Gonal-F; Serono, Switzerland) was injected for the ovarian stimulation. The human chorionic gonadotrophin (hCG; Livzon, Guangdong, China) injections were given when a minimum of 3 follicles developed to reach a size of 18 mm. The serum levels of progesterone (P4), luteinizing hormone (LH), estradiol (E2) and hCG were also detected in the morning on the same day. 34 to 36 h following the hCG administration, oocytes were retrieved using transvaginal ultrasound-guided follicular aspiration. At the same day before oocyte retrieval, serum levels of P4 and E2 were also detected.

### Follicular fluid collection

FF was sampled along with oocytes during the oocyte retrieval procedure. All FF samples were collected in a sterile container upon harvesting of the oocytes. Care was taken to ensure that these FF samples that did not contain flushing solution or blood used for analysis. FF were immediately subjected to a 10-min centrifugation at a speed of 2000 rpm before the product was kept at − 80 °C until time of assay.

### Human granulosa-lutein cell culture

Primary human granulosa cells were extracted and purified from the FF aspirates utilizing density centrifugation as previously described [28]. Dulbecco’s Modified Eagle Medium/nutrient mixture F-12 Ham medium (DMEM/F-12; Gibco, Grand Island, NY) supplemented with 10% charcoal/dextran-treated FBS (HyClone, Logan, UT), 100 μg/mL of streptomycin sulfate and 100 U/mL of penicillin (Boster, China) was used to culture cells at 37 °C in a humidified environment comprising of 95% air and 5% CO_2_. For all of experiments involving melatonin treatments, the cells were plated with 1 mL of culture medium in 12-well plates (5 × 10^4^ cells/cm^2^). The medium was switched to 0.5% charcoal/dextran-treated FBS following a 5-day cultivation period. Melatonin (R&D systems 3550/50, USA) treatments were administered while cells were in this medium. The cells were then left to grow for another 24 h.

### Melatonin ELISA assay

The concentrations of follicular fluid melatonin were determined by melatonin ELISA kits (Abcam213978, UK). All ELISA experiments were run in compliance with the manufacturer’s protocol. Melatonin levels were determined as average levels.

### Reverse transcription quantitative real-time PCR (RT-qPCR)

The RNeasy Plus Mini Kit (QIAGEN, Germany) was utilized to extract total cellular RNA following manufacturer’s protocols. RNA (2 μg) was reverse-transcribed into first-strand cDNA using the GoldScript one-step RT-PCR Kit (Applied Biosystems Grand Island, NY). For qPCR analysis, each sample contained 250 nM of primer, 20 ng of cDNA and 1X SYBR Green PCR Master Mix (Applied Biosystems) to make up a total reaction volume of 20 μL per sample. VEGF, AROM, iNOS and GAPDH gene expressions were determined with TaqMan gene expression assays (Applied Biosystems). Three individual experiments were carried out with various cultures, and assays for each sample were performed at least three times. All qPCR analyses were performed on the a 96-well optical reaction plate Applied Biosystems 7500 Real-Time PCR System. qPCR reaction parameters comprised of 2 min at 50 °C, 10 min at 95 °C, followed by forty 15-s long cycles at 95 °C, ending with 1 min at 60 °C. Primer sequences of utilized primers are as follows: 5′-AAC CAT GAA CTT TCT GCT GTC TTG-3′ (sense) and 5′-TTC ACC ACT TCG TGA TGA TTC TG-3′ (antisense) for VEGF, 5′-GAG AAT TCA TGC GAG TCT GGA-3′ (sense) and 5′-CAT TAT GTG GAA CAT ACT TGA GGA-3′ (antisense) for AROM, 5′-ACA AGC CTA CCC CTC CAG AT-3′ (sense) and 5′-TCC CGT CAG TTG GTA GGT TCT G-3′ (antisense) for iNOS , 5′-CGG AAC GCA GGT AAT TTG TT-3′ (sense) and 5′-CCC AGC CGT CAT AGA AGA TG-3′ (antisense) for MT2 and 5′-ATG GAA ATC CCA TCA CCA TCT T-3′ (sense) and 5′-CGC CCC ACT TGA TTT TGG-3′ (antisense) for GAPDH. PCR products were electrophoresed and a dissociation curve analyses were carried out to validate each assay’s specificity. Efficiency of each assay was confirmed by determining the amplification efficiency with calibration curves to confirm that the log input amount vs. Ct value plot had a slope < |0.1|. The comparative Ct (2 − ΔΔCt) method was used to obtain mean mRNA values with values standardized against expression of the GAPDH gene. All experiments were carried out with at least three repeats with the respective cDNA samples.

### Western blot

The PBS was used to wash cells. Then cells were harvested in cell lysis buffer (Cell Signaling Technology). Equal amounts of protein were transferred onto PVDF membranes after separating by SDS polyacrylamide gel electrophoresis. After 1 h of blocking with 5% non-fat dry milk in Tris-buffered saline + Tween (TBST), the membranes were incubated overnight at 4 °C with anti-melatonin receptor 1B antibody (Abcam203346, UK) and anti-α-tubulin antibody (AT0007, WI), which were diluted in 5% non-fat milk/TBST. Following primary antibody incubation, the membranes were incubated with the anti-rabbit (Abcam6721, UK) or anti-mouse (Abcam97023, UK) HRP-conjugated secondary antibody. Immunoreactive bands were visualized by an enhanced chemiluminescent substrate (Bio-Rad Laboratories; Shanghai, China). The chemiluminescent blots were imaged by the ChemiDoc MP Imager (Bio-Rad Laboratories).

### Statistical analysis

All data analysis was performed using the SPSS version 21.0 statistical program, with a *p* value of less than 0.05 deemed to confer statistical significance. Mean ± standard deviation (SD) was used to present continuous variables that were normally distributed while median (interquartile range) values were used to present variables with highly skewed distribution. One-way ANOVA test, Kruskal–Wallis test and the Mann–Whitney U test were applied onto the data to uncover differences between two groups. The association between FF melatonin concentration and other parameters was measured and tested by Spearman rank correlation coefficients (*r*s). Receiver-operating characteristic (ROC) curves depicting predicted probabilities were generated from logistic regression models of OHSS. The likelihood of OHSS was calculated based on each logistic regression model, which then allowed us to plot stratified ROC curves. Comparisons between ROCs and the area under curves (AUCs) of the models allowed us to determine the presence of differences that were statistically significant.

## Results

### Baseline information of included patients

Basic parameters of two groups are described in Table 1. No significant difference was found in age, BMI, antral follicle count (AFC), basal follicle stimulating hormone (FSH), E2, P4, prolactin (PRL) and testosterone (T) levels as well as duration of infertility between OHSS and non-OHSS groups. The starting dose of gonadotrophin, the gonadotrophin duration and total dose of gonadotrophin were not significant different between two groups. Although higher LH levels were found in OHSS group when compared with non-OHSS patients, both of them were in the normal range (6.81 ± 5.07 vs 4.26 ± 1.67 mIU/ml, *p* = 0.020). It is interesting that the serum E2 and P4 on the hCG day (7667.46 ± 2821.10 vs 4076.58 ± 2343.74 pg/ml, *p* < 0.001 & 1.10 ± 0.42 vs 0.69 ± 0.33 ng/ml, *p* < 0.001) and on OPU day (4306.81 ± 1683.73 vs 2402.39 ± 1666.98 pg/ml, *p* < 0.001 & 22.65 (11.97–34.61) vs 7.28 (5.37–12.08) ng/ml, *p* < 0.001) were significant higher in OHSS group. There was no difference of the number of oocytes that larger than 18 mm before injection between two groups. As expected, the number of total oocytes retrieved (20.46 ± 5.83 vs10.36 ± 4.87, *p* < 0.001), the number of 2PN oocytes (14.15 ± 5.49 vs 7.58 ± 4.07, *p* < 0.001), 2PN cleavage embryos (13.65 ± 5.11 vs 7.48 ± 4.12, *p* < 0.001), total embryos obtained (7.15 ± 2.80 vs 4.07 ± 2.53, p < 0.001) and high-quality embryos obtained (6.31 ± 3.00 vs 3.45 ± 2.50, *p* < 0.001) were also significantly higher in OHSS group. While the ratios of these parameters have no differences between two groups.

**Table 1:** Comparison of clinical characteristics and IVF outcome between the OHSS and non-OHSS groups

Variable	OHSS group (*n* = 26)	Non-OHSS group (*n* = 31)	*P* value
Age of patients (y)	29.39 ± 2.56	28.94 ± 4.04	0.613
BMI (kg/m^2^)	23.25 ± 2.80	23.40 ± 2.91	0.842
Infertility duration(y)	3.62 ± 2.61	3.81 ± 2.66	0.786
Basal serum FSH (mIU/ml)	6.20 ± 1.10	6.93 ± 1.76	0.074
Basal serum LH (mIU/ml)	6.81 ± 5.07	4.26 ± 1.67	0.020^a^
Basal serum E2 (pg/ml)	29.45(21.39–34.61)	25.02(13.35–39.62)	0.671
Basal serum P4 (ng/ml)	0.57(0.38–0.79)	0.54(0.38–0.67)	0.414
Basal serum PRL (ng/ml)	18.24 ± 7.56	18.21 ± 7.94	0.986
Basal serum T (ng/ml)	0.31 ± 0.16	0.25 ± 0.13	0.279
Antral follicle count(n.)	16.89 ± 4.97	13.26 ± 4.75	0.146
Starting dose of Gonadotrophin (IU)	150(112.5–150)	150(112.5–225)	0.174
Gonadotrophin duration (d)	11.54 ± 1.30	10.77 ± 1.91	0.089
Total Gonadotrophin dose (IU)	1425.00(1190.63–1678.13)	1500(1275.00–2125.00)	0.248
Serum E2 on hCG day (pg/ml)	7667.46 ± 2821.10	4076.58 ± 2343.74	<0.001^a^
Serum P4 on hCG day (ng/ml)	1.10 ± 0.42	0.69 ± 0.33	<0.001^a^
Serum E2 on OPU day (pg/ml)	4306.81 ± 1683.73	2402.39 ± 1666.98	<0.001 ^a^
Serum P4 on OPU day (ng/ml)	22.65(11.97–34.61)	7.28(5.37–12.08)	<0.001 ^a^
Larger than 18 mm before injection (n.)	4(3–5)	4(3–5.25)	0.201
Total oocytes obtained (n.)	20.46 ± 5.83	10.36 ± 4.87	<0.001^a^
No. of 2PN oocytes	14.15 ± 5.49	7.58 ± 4.07	<0.001^a^
No. of 2PN cleavage embryos	13.65 ± 5.11	7.48 ± 4.12	<0.001^a^
High-quality embryos (n.)	6.31 ± 3.00	3.45 ± 2.50	<0.001 ^a^
2PN oocytes ratio	0.74 ± 0.04	0.69 ± 0.03	0.351
2PN cleavage embryos ratio	0.72 ± 0.04	0.67 ± 0.03	0.32
Total embryos ratio	0.37 ± 0.03	0.42 ± 0.04	0.245
High-quality embryos ratio	0.35 ± 0.04	0.32 ± 0.03	0.596
FF melatonin(ng/ml)	35.94 ± 10.18	23.93 ± 10.94	<0.001^a^

^a^*P* < 0.05 was considered statistically significant

### Patients in the OHSS group showed significantly higher melatonin concentrations which correlated positively with epidemiological parameters

**Fig. 1:**
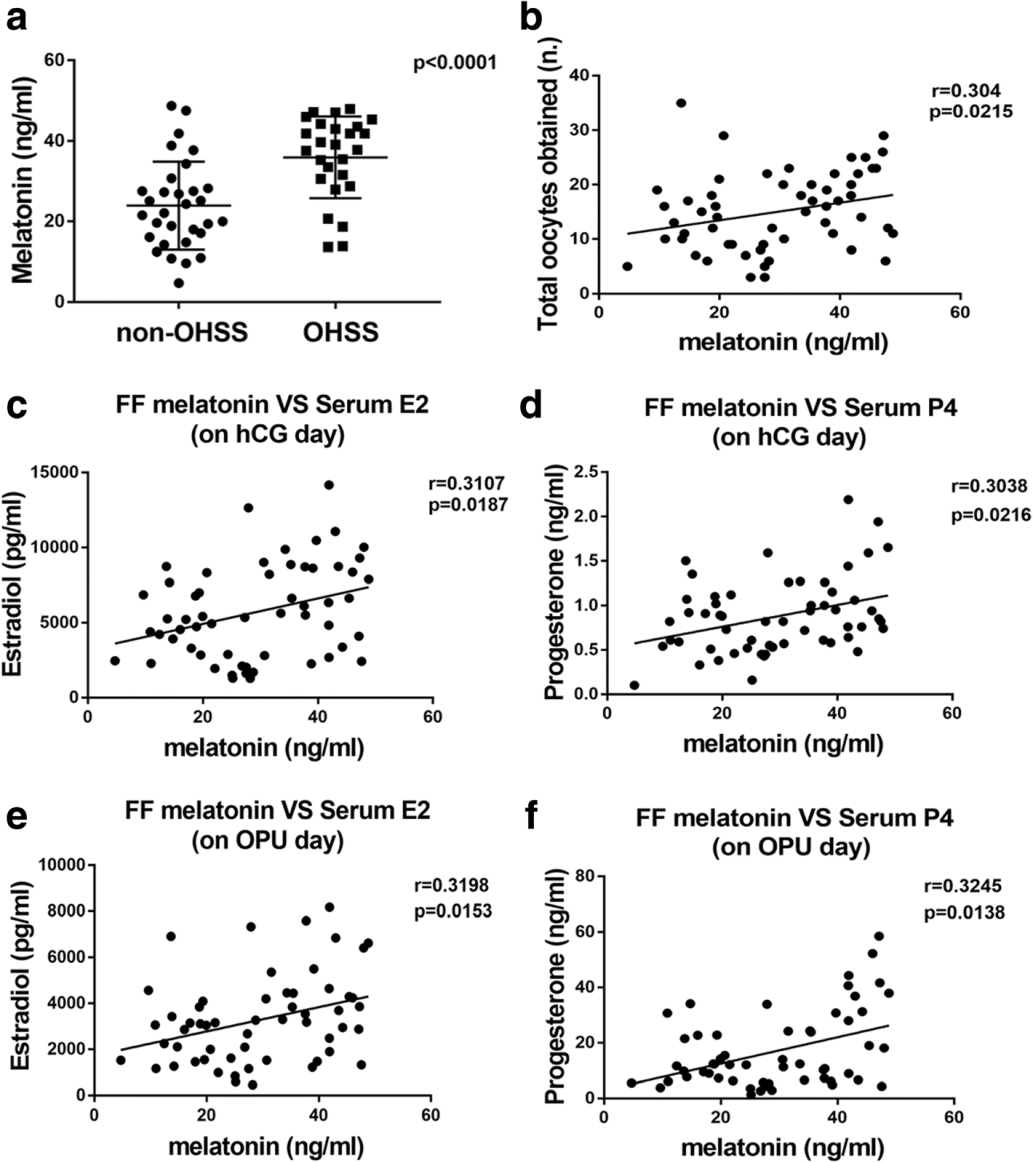
The difference of melatonin between OHSS and non-OHSS groups and the correlation with epidemiological parameters. (**a**): The difference of FF melatonin between the OHSS and non-OHSS groups(*p* < 0.0001). (**b**): Correlation between FF melatonin and the number of total oocytes obtained (r = 0.304; *p* = 0.0215). (**c**): Correlation between FF melatonin and serum E2 on the day of hCG administration (r = 0.3107; *p* = 0.0187). (**d**): Correlation between FF melatonin and serum P4 on the day of hCG administration (r = 0.3038; *p* = 0.0216). (**e**): Correlation between FF melatonin and serum E2 on OPU day (r = 0.3198; *p* = 0.0153). (**f**): Correlation between FF melatonin and serum P4 on OPU day (r = 0.3245; *p* = 0.0138)

As shown in Table 1 and Fig. 1A, the FF melatonin concentrations on OPU day in the OHSS group were higher when compared to those in the non-OHSS group (35.94 ± 10.18 ng/mL vs 23.93 ± 10.94 ng/mL, *p*<0.001). Figure 1 presents the correlations between FF melatonin concentrations and clinical characteristics of both non-OHSS and OHSS patients. The FF melatonin concentrations were significantly positively correlated with the total number of oocytes retrieved (r = 0.304, *p* = 0.0215, Fig. 1B), serum E2 levels (r = 0.3107, *p* = 0.0187, Fig. 1C), and serum P4 levels (r = 0.3038, *p* = 0.0216, Fig. 1D) on the day that patients were given hCG as well as serum E2 levels (r = 0.3198, *p* = 0.0153, Fig. 1E), and serum P4 levels (r = 0.3245, *p* = 0.0138, Fig. 1F) on OPU day.

### Patients in the OHSS group displayed significantly higher MT2 expression

**Fig. 2:**
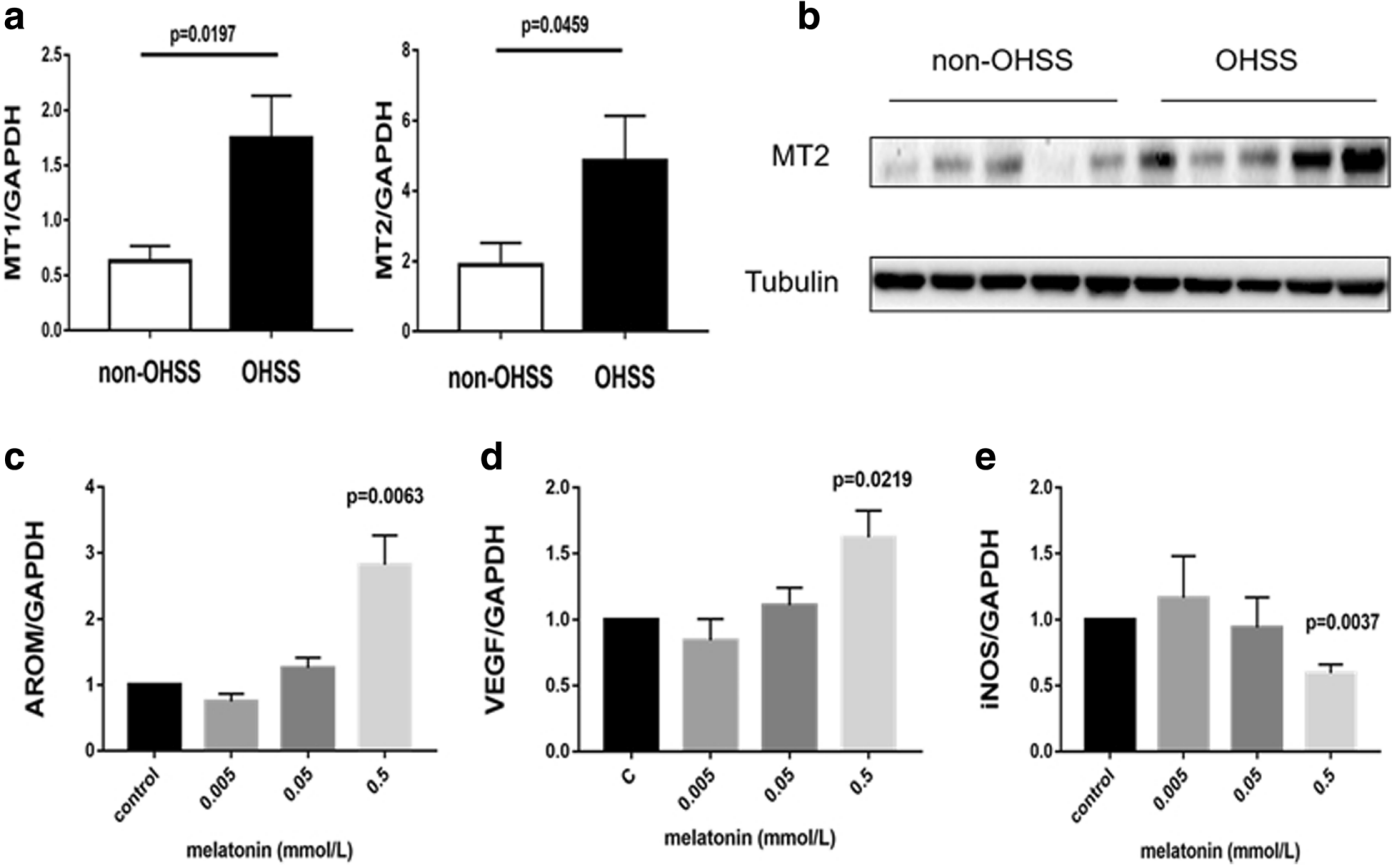
Higher MT2 were found in OHSS patients and melatonin mediates AROM, VEGF and iNOS expression. Granulosa cells were collected from the OHSS patients and the non-OHSS patients. (**a**):MT2 mRNA expression was significantly higher in the OHSS group (*p* = 0.0459). (**b**):MT2 protein expression was significantly higher in the OHSS group. Granulosa cells of non-OHSS patients were treated with 0.005, 0.05 and 0.5 mmol/L of melatonin for 24 h. (**c**): melatonin of 0.5 mmol/L up-regulated AROM mRNA expression (*p* < 0.0063). (**d**): melatonin of 0.5 mmol/L up-regulated VEGF mRNA expression (*p* = 0.0219). (**e**): melatonin of 0.5 mmol/L down-regulated iNOS mRNA expression (*p* = 0.0037)

Human granulosa cells were collected from OHSS and non-OHSS patients and the levels of MT2 mRNA and protein expression were determined by RT-qPCR and western blot respectively. As shown in Fig. 2A and B, the MT2 mRNA was notably higher in the OHSS group (*p* = 0.0459) as well as the protein expression.

### Melatonin up-regulates aromatase mRNA and VEGF mRNA expression and down-regulates iNOS mRNA expression

We treated human granulosa cells with 0.005, 0.05 or 0.5 mmol/L melatonin for 24 h, then we measured aromatase, VEGF and iNOS mRNA expression. As shown in Fig. 2, the result indicated that 0.005 or 0.05 mmol/L melatonin did not affect aromatase, VEGF and iNOS mRNA expression, whereas treatment with 0.5 mmol/L melatonin could significantly up-regulated aromatase (Fig. 2C) mRNA and VEGF (Fig. 2D) mRNA expression while decreasing iNOS (Fig. 2E) mRNA in human granulosa cells.

### The cut-off level of melatonin for the prediction of the diagnosis of OHSS

**Fig. 3:**
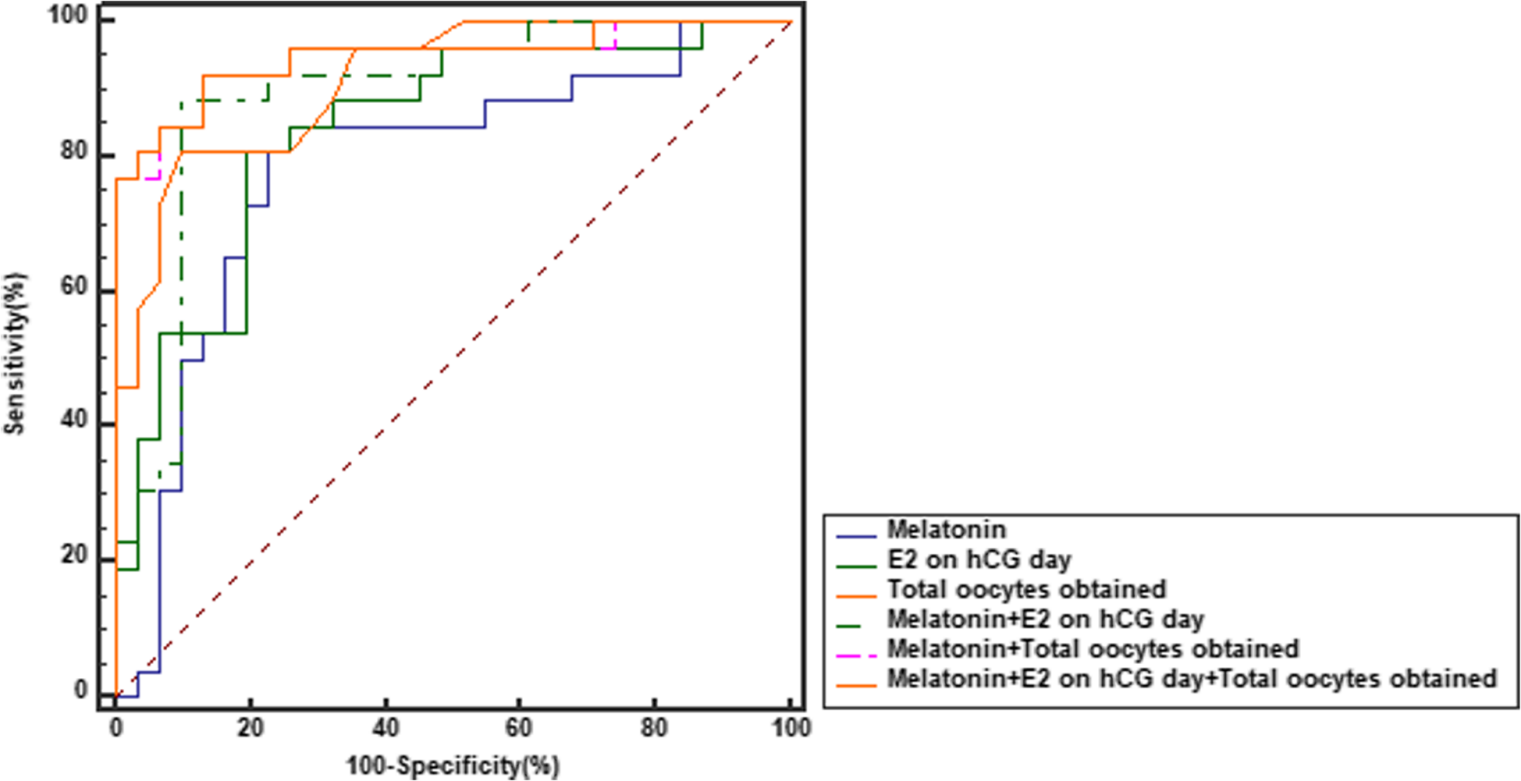
Comparison of ROC curves. Melatonin in combination with the E2 level on hCG day and the number of total embryos obtained have a bigger AUC than other parameters

Figure 3 and Table 2 both depict the sensitivity and specificity values as predicted by ROC curve analyses. Using a melatonin cut-off level of 27.52 ng/ml, the sensitivity and specificity for melatonin to predict OHSS were 84.6 and 74.0%, respectively (*p* < 0.0001). We further determined a multivariable method for the prediction of OHSS. Our result showed that a model combining melatonin concentration with the E2 level on hCG day and the number of total oocytes obtained get a larger AUC than melatonin (0.949 vs 0.785, *p* = 0.0046) and serum E2 level on hCG day (0.949 vs 0.840, *p* = 0.0297), as well as the total oocytes obtained (0.949 vs 0.914, *p* = 0.1798), alone.

**Table 2:** The sensitivity and specificity values of ROC curve

Variable	Cut-off	Sensitivity(%)	Specificity(%)	AUC(CI)	*P*
Melatonin (ng/ml)	> 27.52	84.6	74.2	0.785(0.657–0.883)	< 0.0001
E2 on hCG (pg/ml)	> 5420	80.77	80.65	0.840(0.719–0.924)	< 0.0001
Total oocytes obtained (n.)	> 16	80.8	90.3	0.914(0.809–0.972)	< 0.0001
Melatonin and E2 on hCG	–	88.5	90.3	0.892(0.782–0.959)	< 0.0001
Melatonin and Total oocytes obtained	–	92.3	87.1	0.947(0.853–0.989)	< 0.0001
Melatonin, E2 on hCG and Total oocytes obtained	–	92.3	87.1	0.949(0.856–0.990)	< 0.0001

*AUC* area under curve, *CI* confidence interval

## Discussion

COH is one of the most important processes in the assisted reproductive technology. During this process, a certain degree of ovarian stimulation is desirable as it can obtain more oocytes. In our study, the OHSS group presented a better outcome of the number of the oocytes and the embryos. However, the ratios of these parameters have no statistical differences. A prospective clinical trial showed that although the differences did not reach statistical significance, the clinical pregnancy rate and implantation rate were in a high tendency in the group treated with melatonin [29]. Thus, it is difficult for us to draw a conclusion whether the high FF melatonin concentration is a positive or a negative criterium for the IVF/ICSI outcome. In our study, we compared the FF melatonin concentrations between OHSS and non-OHSS patients and we found that OHSS patients tend to have higher FF melatonin levels. Moreover, the FF melatonin concentrations were found to correlate positively with concentrations of serum E2 and serum P4 both on the hCG day and OPU day. Patients with higher FF melatonin concentrations also retrieved more numbers of oocytes on OPU day. All of these parameters are important indexes to predict the occurrence of OHSS [30]. Thus, the concentration of melatonin in the FF might also be a predictor of OHSS. The ROC curves in our study indicates the potential clinical value of melatonin in predicting OHSS as well.

Ovarian response and follicle fluid microenvironment are reported to be directly correlated with the risk of OHSS. As a biological window during the maturation of oocytes, follicular fluid contains cytokines, hormones, oxidation/antioxidant systems and various metabolites. They form a complex regulatory system to affect the process of OHSS [31]. Interestingly, existing literature shows that the concentration of melatonin in FF is more than three times higher than that in serum [23]. In addition, it was reported that melatonin could improve the maturation and quantity of oocytes [21, 32]. Patients with high ovarian response also had high melatonin concentration [26].

As the largest cell population in the follicle, granulosa cells regulate the growth, development and maturation of follicles in an autocrine and paracrine manner [33]. MT2 has been shown to be closely linked to PCOS and vascular permeability [9, 11]. In our study, we found that the OHSS patients had higher MT2 expression level in human granulosa cells. This further indicated that melatonin and its receptor were involved in the regulation of OHSS. One of the molecular pathogenesis that melatonin may involve is by increasing aromatase levels to accelerate the conversion of androgen to estrogen in human granulosa cells. Our clinical data which showed that melatonin FF concentrations positively correlated with serum E2 levels measured on the day of hCG injection as well as the OPU day also supported this point. It was widely accepted that both high level of VEGF and low level of NO can increase the permeability of cell membrane, resulting in the occurrence of OHSS [34, 35]. Previous studies have also confirmed NO can inhibit the production of estrogen, and iNOS can regulate vascular immune inflammation [20, 36]. As expected, our results showed that melatonin can up-regulate VEGF expression and down-regulate iNOS expression respectively in the human granulosa cells. This indicated that melatonin probably increased the vascular permeability, leading to the extravasation of body fluids which are responsible for the symptoms observed in OHSS. The regulatory role of melatonin to cells dependents on concentration and cell type [37]. For example, 1 mM melatonin can significantly up-regulated the mRNA expression of C/EBPβ and C/EBPα in bovine intramuscular preadipocytes (BIPs). While lower melatonin doses were inefficient [38]. In our study, the significant effects of melatonin on aromatase, VEGF and iNOS mRNA expression were only observed when cells were treated with the 0.5 mmol/L melatonin. Thus the effect of melatonin in vitro may be specific for different types of cells. However, further studies are required to confirm this hypothesis.

The early prevention and diagnosis of OHSS is still a big challenge in COH process. Presently, several parameters with high sensitivity and specificity for the prediction of OHSS have been identified [39], such as serum E2 concentrations on the day when hCG is injected and the total number of oocytes obtained [40]. In our study, we firstly demonstrated that FF melatonin concentration could be used as a predictor for the occurrence of OHSS. Especially, its predictive power increases when combined with the E2 level on hCG administration day and the number of total oocytes obtained. Our study not only suggests that melatonin may have clinical benefits in the prediction of OHSS, but also indicate that the melatonin can be used as a target for OHSS treatment.

## Conclusion

In our study, we demonstrated for the first time the concentrations of melatonin in follicle fluid are related to the OHSS and could be used as a potential predictor of the OHSS. Furthermore, our results showed treatment with melatonin can regulate the associated factors expression of OHSS. Our study showed melatonin may participate in the occurrence of OHSS. However, further large-scale and prospective investigation are required to validate the use of melatonin in the prediction and prevention of OHSS establishment.

## Abbreviations

AFC: antral follicle count
aromatase: P450 aromatase cytochrome
AUCs: area under curves
COH: controlled ovarian hyperstimulation
E2: estradiol
FF: follicle fluid
FSH: follicle stimulating hormone
iNOS: inducible nitric oxide synthase
LH: luteinizing hormone
MT1: melatonin receptor 1
MT2: melatonin receptor 2
NO: nitric oxide
OHSS: Ovarian hyperstimulation syndrome
P4: progesterone
PCOS: polycystic ovarian syndrome
PRL: prolactin
r: rank correlation coefficients
RAS: rennin angiotensinogen system
ROC: receiver-operating characteristic
RT-qPCR: Reverse transcription quantitative real-time PCR
SD: standard deviation
SNP: single-nucleotide polymorphisms
T: testosterone
TNF: tumor necrosis factor
VEGF: vascular endothelial growth factor
